# Personalized nutrition, microbiota, and metabolism: A triad for eudaimonia

**DOI:** 10.3389/fmolb.2022.1038830

**Published:** 2022-10-18

**Authors:** Muhammad Hassan Sarfraz, Aqsa Shahid, Samra Asghar, Bilal Aslam, Usman Ali Ashfaq, Hammad Raza, Miguel A. Prieto, Jesus Simal-Gandara, Francisco J. Barba, Muhammad Shahid Riaz Rajoka, Mohsin Khurshid, Abdulqadir J. Nashwan

**Affiliations:** ^1^ Department of Microbiology, Government College University Faisalabad, Faisalabad, Pakistan; ^2^ Faculty of Rehabilitation and Allied Health Sciences, Riphah International University, Faisalabad, Pakistan; ^3^ Department of Bioinformatics and Biotechnology, Government College University Faisalabad, Faisalabad, Pakistan; ^4^ Department of Biochemistry, Government College University Faisalabad, Faisalabad, Pakistan; ^5^ Nutrition and Bromatology Group, Faculty of Food Science and Technology, University of Vigo, Ourense, Spain; ^6^ Centro de Investigação de Montanha (CIMO), Instituto Politécnico de Bragança, Bragança, Portugal; ^7^ Nutrition and Food Science Area, Preventive Medicine and Public Health, Food Science, Toxicology and Forensic Medicine Department, Faculty of Pharmacy, Universitat de València, Burjassot, València, Spain; ^8^ Food and Feed Immunology Group, Graduate School of Agricultural Science, Tohoku University, Sendai, Japan; ^9^ Nursing Department, Hazm Mebaireek General Hospital (HMGH), Hamad Medical Corporation (HMC), Doha, Qatar

**Keywords:** personalized nutrition, microbiota, metabolism, diet, digestion, immunity, nutrition, genetics

## Abstract

During the previous few years, the relationship between the gut microbiota, metabolic disorders, and diet has come to light, especially due to the understanding of the mechanisms that particularly link the gut microbiota with obesity in animal models and clinical trials. Research has led to the understanding that the responses of individuals to dietary inputs vary remarkably therefore no single diet can be suggested to every individual. The variations are attributed to differences in the microbiome and host characteristics. In general, it is believed that the immanent nature of host-derived factors makes them difficult to modulate. However, diet can more easily shape the microbiome, potentially influencing human physiology through modulation of digestion, absorption, mucosal immune response, and the availability of bioactive compounds. Thus, diet could be useful to influence the physiology of the host, as well as to ameliorate various disorders. In the present study, we have described recent developments in understanding the disparities of gut microbiota populations between individuals and the primary role of diet-microbiota interactions in modulating human physiology. A deeper understanding of these relationships can be useful for proposing personalized nutrition strategies and nutrition-based therapeutic interventions to improve human health.

## Introduction

The three biologically and chemically complex systems work together namely the diet, the gut microbiota, and the host’s metabolisms are interconnected. The diet, consisting of many different molecules, varies greatly among individuals in terms of composition and consumption habits. The microbiota is composed of hundreds of microbial species in a symbiotic relationship with the human host and is of vital importance to the host’s health. The mode of birth (vaginal or cesarean section) and diet are the early events that impact the gut microbiota. Diet is a crucial factor in the configuration of the gut microbiota and can modulate the abundance of microbial species and their functions (David et al., 2014; Leeming et al., 2019). Finally, host metabolism involves biological molecules, digestive enzymes, and mucosal immune regulation (Kau et al., 2011; Zmora et al., 2019).

Several studies have suggested that modulation of the host response to dietary components by the gut microbial species could influences metabolism, the precise underlying mechanisms are remarkably complex. It has been reported that the microbiota controls the pathogenesis and progression of various metabolic disorders and can also influence the treatment of diseases (Cho and Blaser, 2012; Parekh et al., 2014). For example, emerging evidence has linked reduced microbial diversity with obesity (Lone et al., 2018). Furthermore, microbial metabolites such as short-chain fatty acids (SCFAs) can affect host physiology. The influence of the environment on the microbiome is greater compared to the effect of the genetic characteristics of the host, and therefore the microbiome is more susceptible to various alterations (Arrieta et al., 2014; Laursen et al., 2017; Rothschild et al., 2018). Therefore, the microbiota is an attractive target for dietary intervention, as it can be modified relatively easily in terms of composition and general functions (Khan et al., 2019; Leeming et al., 2019; Liu et al., 2020). In this sense, personalized nutrition is increasingly recognized as a new therapeutic pathway that can modulate host-microbiota interactions to prevent and control metabolic disorders.

The main challenge of personalized nutrition is the identification of important characteristics of the human microbiome that could help in the prediction of the metabolic response of the host to dietary components that may be useful in designing personalized diets with favorable results. For this personalized approach, it is necessary to interrelate the microbiome, dietary treatment, and host response. In this review, we have summarized recent concepts for a better understanding of the main role of gut microbiota in metabolism. In addition, the impact of diet on the structure and function of the microbiota and the role of the diet-microbiome interaction in the development of certain metabolic diseases could help to outline the concepts for designing personalized diets.

## Diversity of gut microbiome across the population

**FIGURE 1:**
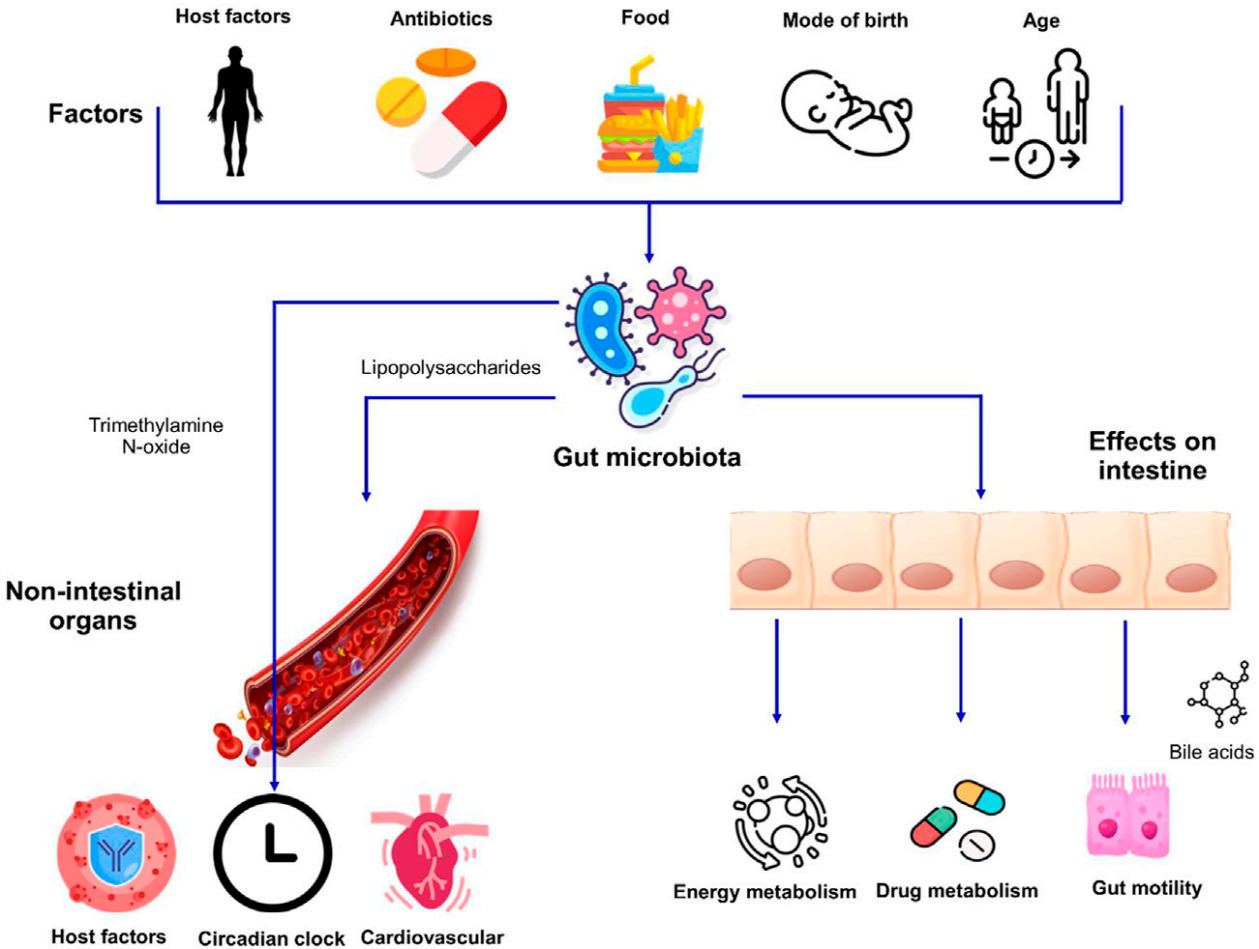
Factors affecting the gut microbiota and its impact on host physiology. Host factors i.e., genetics and age as well as exogenous factors can influence the makeup of microbiota. The interactions between the intestinal microbiome and dietary habits result in alterations in different aspects of human physiology affecting both the intestinal and non-intestinal organs.

The environment, diet, immune system, the use of antimicrobial agents, medications, hygiene, and climatic conditions are some of the prominent factors driving variations in the gut microbiota between individuals (Anwar et al., 2021) (Figure 1). The gut microbiota starts seeding during birth and largely develops during the first 3 years of life (Koenig et al., 2011). The neonates get exposed to microbes from different sources and therefore the initial colonization of their intestinal tract is mainly dependent on the microbial species being encountered. The mode of delivery also impacts the initial population diversity as the vaginally delivered babies harbor the microbiota that resembles the microbial communities present in the vaginal tract of their mother whereas babies born through Caesarean section usually acquire the microbes from the skin of their mother or the caretakers and is dominated by the taxa, for example, Staphylococcus and Propionibacterium (Dominguez-Bello et al., 2010).

The mode of feeding also influences the gut microbiota in infants as the breast milk normally contains maternal antibodies, nutrients, and diverse commensal bacteria such as lactobacilli and bifidobacteria. Therefore, breastfed infants have higher level of lower levels of Bifidobacterium and lower levels of Atopobium compared to formula-fed infants. Initially, the diversity of gut microflora in babies is low and increases with the developmental stages. At around 3 years of life, the microbial community composition becomes like an adult. The major shifts in the microbial species are mainly associated with the intake of solid food (Koenig et al., 2011; Jost et al., 2013).

### Influence of lifestyle on microbiome structure

The diet is a major and well-recognized determinant that can answer the distinctions in microbial composition between individuals (Leeming et al., 2019; Moles and Otaegui, 2020). The main evidence for the influence of diet on the human gut microbiota comes from traditional (hunter-gatherer) societies that experience seasonal variations in their diet. For example, the study conducted in the Hadza tribal community in Tanzania showed that the relative abundance of Bacteroidetes was lower in the wet season due to the higher consumption of berries and honey compared to the dry season, the hunting season (Smits et al., 2017). The North American Hutterite population consumes canned or frozen foods in winter and more fresh fruits and vegetables during the summer season, which is believed to play a role in the differences in the microbial composition of their feces between these two seasons. Bacteroidetes are more abundant in the summer to digest the complex carbohydrates provided by the diet holding more fiber, while Actinobacteria are depleted (Davenport et al., 2014).

The environment is another crucial factor that leads to changes in diet and consequent microbial populations, as it is associated with the loss of diversity and the decline or loss of particular microbial species (Obregon-Tito et al., 2015). Consumption of raw or wild foods by non-urbanized populations, for example, the Hadza community, results in a more diverse gut ecosystem, compared to urban populations that primarily consume a diet consisting of commercial or processed agricultural food products (Schnorr et al., 2014; Ayeni et al., 2018a). The higher fiber diet of the rural population results in the enrichment of Bacteroidetes, especially genus *Xylanibacter* and *Prevotella*, allowing the host to make the best use of dietary fibers (Senghor et al., 2018). In another study, the loss of microbial diversity in westernized people, from developing countries who settled in the United States, was reported. The genus *Bacteroides* began to replace *Prevotella* in the gut of these immigrants, showing the replacement of non-western-associated microbial species with western-associated species (Vangay et al., 2018). However, the homogeneous and simpler diet of the population living in rural areas compared to the greater variety of food intake by the urbanized population leads to greater variability of the gut microbiomes among the urbanized population (Ayeni et al., 2018b; Das et al., 2018). Furthermore, variations in eating habits and lifestyles, such as improved hygiene, contamination, and the use of antibiotics may contribute to greater variability of the gut population in urbanized societies (Kolodziejczyk et al., 2019).

### Influence of diet on the intestinal microbiota

**FIGURE 2:**
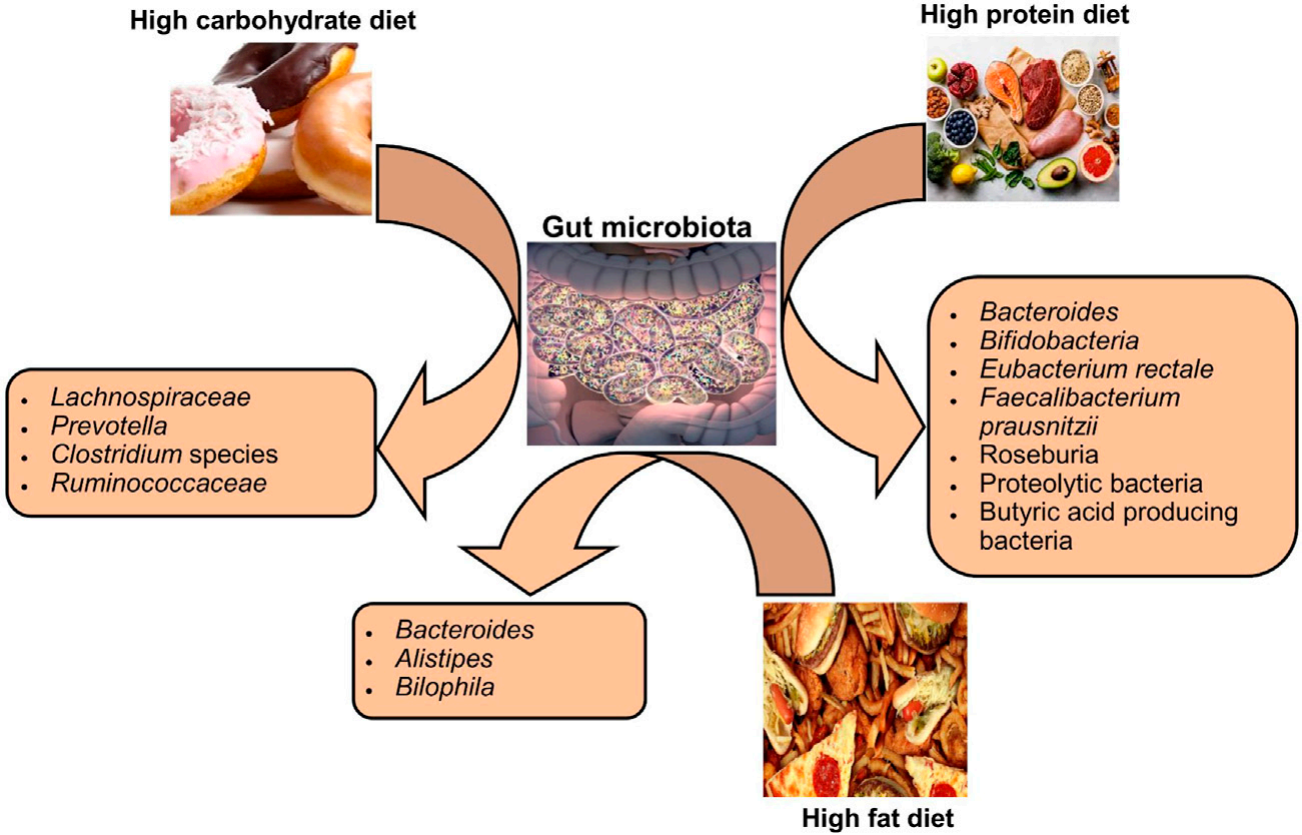
Impact of diet types on the composition of gut microbiota.

Variations in dietary macronutrients, including proteins, carbohydrates, and fats, can cause substantial alterations in the gut microbial population (David et al., 2014; Sun et al., 2022). The dysbiosis of gut microbiota including decreased stability, reduced diversity, and relative abundance of certain bacteria as shown in Figure 2. Studies in humans have shown that diet-induced variations in the gut microbiota tend to occur rapidly (Table 1). For example, the change from an omnivorous diet to a vegetarian diet shows substantial changes in the intestinal microbiota in 4 days, while the variation in the consumption of the type of fats or dietary fiber is reflected in 14 days (Tomova et al., 2019; Losno et al., 2021). However, minor modifications, such as consuming different types of bread, resulted in a minor change in the composition of the gut microbiota between individuals (Korem et al., 2017). In particular, the inter-individual variability of the gut microbiota is controlled by many other factors, such as gender, age, ethnicity, and medications, in addition to the diet (Brooks et al., 2018; Rothschild et al., 2018).

**TABLE 1:** The response of gut microbiome to the dietary interventions.

Dietary interventions	Duration	Study design	Sample size (population)	Population types	Analysis platform	Outcomes	References
No fiber, polydextrose (21 g/day); soluble corn fiber (21 g/day)	21 days	A randomized double-blind, placebo-controlled crossover trial	20	Normal weight (males)	Pyrosequencing 16S rRNA gene	A shift in the gut microbial community with fiber interventions	Hooda et al. (2012)
Whole grain 150 g/day) verses refined grain	14 days	Longitudinal crossover study	17	Normal weight individuals	FISH for bacterial species enumeration	An increase in the *Clostridium leptum* group with the whole grain diet	Ross et al. (2011)
130 g of pinto bean (dried and cooked) chicken-noodle soup	12 weeks	Factorial design	80 (40 each in 2 groups)	Normal weight pre-metabolic syndrome and control	FISH for bacterial species enumeration	*Eubacterium limosum* levels were decreased by 50% in the beans group	Finley et al. (2007)
Controlled basal diet fortified with either High resistant Starch (amylomaize starch) or Low resistant Starch (cornstarch)	04 weeks	Randomized controlled crossover	12	Normal weight individuals		Differences in breath hydrogen levels. Changes in the fecal parameters (pH, β-glucosidase, certain SCFAs, secondary bile acids) associated with bacterial activity	Hylla et al. (1998)
Fructo-oligosaccharides (20 g) and 10 g partially hydrolyzed guar gum (10 g) per day vs. placebo	21 days	Human volunteer study	31	Normal weight	FISH for bacterial species enumeration	Increase in Bifidobacterium species No change in fecal pH	Tuohy et al. (2001)
A high-fiber diet containing LKFiber compared to a control diet	01 month	A single-blind, randomized, crossover study	38	Normal weight males	Short chain fatty acids and bacterial enzymatic activity in feces	Higher fiber altered the parameters of bowel function, decreased fecal pH., Increased fecal SCFA and decreased β-glucuronidase activity	Johnson et al. (2006)
48 g breakfast cereals, (whole grain or wheat bran)	03 weeks periods (Twice)	A double-blind, placebo-controlled, crossover study	31	Normal weight individuals	FISH for the enumeration of bacterial groups	The numbers of fecal Bifidobacteria and lactobacilli were substantially higher in the whole-grain group. Significant increase in serum ferulic acid levels	Costabile et al. (2008)
LKFiber diet containing additional 17–30-g fiber per day compared with a control diet	28 days	Single-blind, randomized, crossover	18	Healthy males	Quantitative FISH analysis	Significantly higher levels of Bifidobacterium species and lower clostridial species in the LKFibre diet group	Smith et al. (2006)
Reduced-fat diet or reduced-carbohydrate diet	01 year	Clinical trial	12	Obese individuals	Pyrosequencing of 16S rRNA gene	The relative abundance of Bacteroidetes increased while the Firmicutes were decreased over time but were not associated with the diet type	Ley et al. (2006)
High protein and medium carbohydrate (164 g/day) compared with high protein and low carbohydrate (24 g/day)	04 weeks	Randomized controlled crossover	19	Obese individuals	FISH for major bacterial groups enumeration	Certain groups of bacteria varied with the diet type. The short-chain fatty acids differed by the diet type	Duncan et al. (2007)
Maintenance diet (360 g carbohydrate, 116 g fat, and 85 g protein per day). High-protein (139 g protein and moderate-carbohydrate (181 g carbohydrate) diet, and 82 g fat/day) and a high-protein (137 g protein, low-carbohydrate (22 g carbohydrate) and 143 g fat/day diet	07 days maintenance diet followed by 04 weeks intervention	Randomized controlled crossover	17	Obese males	FISH for bacterial 16S rRNA genes	The high protein and low carbohydrate diet decreased the population of *Eubacterium rectale* and Roseburia genus	Russell et al. (2011)
High (43 g/day) cereal fiber, or control (14 g/day) cereal fiber, or high-protein (28% of energy-intake) along with 14 g/day cereal-fiber, or moderately high cereal fiber (26 g/day) with protein (23% of energy-intake)	18 weeks	Randomized controlled crossover	69	overweight Individuals	FISH for bacterial groups enumeration	No effect of diet on the population of gut bacteria	Weickert et al. (2011)

The protein content of foods has a variable influence on the composition and relative abundance of intestinal microbial species among individuals (Zhu et al., 2015). Protein sources (meat or non-meat) are known to alter the gut microbiota in rats (Zhu et al., 2015). The consumption of a diet rich in animal proteins is associated with a higher number of *Bacteroides* in humans, even in the short term it increases bile-tolerant species such as *Bilophila* and *Alistipes* and decreases saccharolytic bacteria such as *Roseburia* species, *Eubacterium rectole,* and *Ruminococcus bromii* (Wu et al., 2011; David et al., 2014). On the other hand, the intake of a diet rich in plant proteins increased the population of Bifidobacteria and Lactobacilli and increased the production of SCFAs in humans (Markowiak-Kopeć and Śliżewska, 2020). The extent of gut microbiota changes in healthy human subjects in response to the consumption of protein from different sources, that is, white meat, red meat, and non-meat sources, is quite variable between different individuals, even in the same population (Lang et al., 2018).

The impact of carbohydrate consumption on the gut microbiota depends on the types and amount of carbohydrates. Long-term intake of complex carbohydrates promotes *Prevotella* genus in humans (Vinke et al., 2017). Since different types of carbohydrates are energy sources for specific bacteria, the type of carbohydrate in the diet can alter the abundance of a particular species. Bifidobacteria efficiently degrade arabinoxylans from wheat and other grains, so humans who eat a low-gluten diet have less abundance of these bacteria in their intestines (Hansen et al., 2018). A diet rich in indigestible carbohydrates significantly increased species of bacteria belonging to the phylum Firmicutes, such as *E. rectole*, *Roseburia* species, and *Ruminococci* species in overweight individuals (Walker et al., 2011). In contrast, less fermentable carbohydrate diets resulted in a substantial decrease in butyrate-producing Firmicutes in obese individuals (Beam et al., 2021). The lack of dietary fiber in the mouse models stimulated the population of mucus-degrading bacteria, leading to colonic barrier dysfunction and thus increased susceptibility to mucosal pathogens (Desai et al., 2016).

Studies have reported similarities in gut microbial patterns within the population in response to a fiber-containing diet. However, diverse and personalized trends have also been reported in the gut microbial population in response to carbohydrate intakes, such as dietary fiber, starches, and prebiotics (Bouhnik et al., 2004; Walker et al., 2011; Tap et al., 2015; Korem et al., 2017). For example, the intake of a high-fiber diet in overweight individuals alters the relative population of the gut microbiota, although there is significant variation between individuals (Cotillard et al., 2013; Korpela et al., 2014; Salonen et al., 2014). In addition, the consumption of indigestible carbohydrates usually causes an increase in fecal butyrate levels, but differences are seen between the study population (Mcorist et al., 2011). Eating habits before a dietary intervention could affect the intestinal microbiota. For example, the response of the gut microbiota to plant-based carbohydrates (inulin-type fructans) is more pronounced among people who regularly consume a lot of fiber compared to those with a typical low fiber intake (Healey et al., 2018). These studies highlight the importance of habitual dietary patterns in modulating the gut microbiota through any dietary intervention.

The published data have suggested that fat intake affects the relative abundance as well as the function of the gut microbiota, which consequently affects the metabolism of the host. In mice, ingestion of a diet low in fiber and high in saturated fat results in an increase in Proteobacteria and Firmicutes and a decrease in the population of Bacteroidetes (Magne et al., 2020). In mice, a diet high in fat and sucrose increased the percentage of body fat, and it was associated with an increase in *Alobaculum* and *Lactococcus* and a decrease in *Akkermansia* species (Parks et al., 2013). However, the results of association studies in rodents regarding dietary fat intake and microbiome change may not be the same as in humans, possibly due to deviations in the complexity of the diet, metabolic instabilities, and disparities in the complexity of the microbiome between humans and rodents (Lai et al., 2014; Mokkala et al., 2020). A higher intake of fats composed of fatty acids (saturated) is associated with lower diversity in the microbial populations of human beings (Wolters et al., 2019). The role of diverse types of dietary fats diet in tempering the overall composition of the intestinal microbiota has not yet been investigated in detail. The study has suggested that the intake of polyunsaturated fatty acids (PUFAs), especially omega-3 fatty acids, which also have anti-cancer and anti-inflammatory effects, leads to a greater richness of many butyrate-producing bacterial species in healthy subjects (Watson et al., 2018). Alteration of the intestinal bacterial population in response to dietary fat intake is highly person-specific, therefore slight to moderate alteration in the dietary intake of saturated fats can result in markedly inter-individual microbiota response variables in healthy people (Lang et al., 2018).

The consumption of foods with additives for instance emulsifiers and artificial sweeteners can influence the gut microbiota. Studies in animal models and clinical trials have shown that the use of artificial (non-caloric) sweeteners, such as aspartame, saccharin, and sucralose affects the composition of the gut microbiota (Ruiz-Ojeda et al., 2019). Despite these compounds being generally safe and widely used, studies in mouse models have highlighted their role in the development of inflammatory and metabolic disorders by inducing intestinal dysbiosis. For instance, sucralose consumption has been linked with intimal inflammation and saccharin consumption with alterations in lipid metabolism and with inflammation of the liver (Bian et al., 2017; Uebanso et al., 2017; Rodriguez-Palacios et al., 2018). Additionally, glucose intolerance is associated with altered microbial populations, and functions of the intestinal microbiota are suggested (Suez et al., 2014). It is important to consider the personalized responses in humans regarding the intake of artificial sweeteners in myriad studies, perhaps due to differences in gut microbial signatures. However, more studies are desired to confirm such findings. Dietary emulsifiers increased mucolytic bacteria for instance *Ruminococcus gnavus* and reduced the content of Bacteroidales in the gut of mice, which may lead to the development of metabolic syndrome (Chassaing et al., 2015). In a mouse model, low-grade inflammation was seen in response to dietary emulsifiers through increased levels of lipopolysaccharides and flagellin, which can cause colon carcinogenesis (Vangay et al., 2018).

### Probiotics and prebiotics

Probiotics are represented as the most widely used dietary supplements. Though, the role of probiotics in shaping the human gut microbiome is not yet conclusive (Khurshid et al., 2015; Afzal et al., 2020; Khurshid and Akash, 2020; Paray et al., 2020). In most studies, the genus Lactobacillus was used to ameliorate obesity in rodents which lead to various metabolic benefits such as reduction in body fat and adipocyte cell size, and control of unnecessary body weight gain (Luoto et al., 2010; Takemura et al., 2010). The administration of *Lactobacillus gasseri* strain could result in decreased body weight and limit the fat mass gain in obese mice being fed a high-sucrose diet (Kang et al., 2010). Several studies used either the Bifidobacterium strains alone for example, *B. adolescentis* or *B. longum* or a combination of different Bifidobacterium species. Such studies have reported the role of Bifidobacterium species in decreasing the adipose tissue and limiting body weight gain in high-fat diet-induced obese rodents (An et al., 2011; Chen et al., 2012). Few clinical studies also reported the effect of probiotic administration on weight reduction and a decrease in body fat (Kadooka et al., 2010; Jung et al., 2013). The supplementation with prebiotics also decreased the adipocyte size, body weight gain, and insulin resistance in obese rodents (Dewulf et al., 2011; Neyrinck et al., 2011). Further, the role of prebiotics in reducing body weight and improving metabolic parameters i.e., insulin resistance was also assessed in obese individuals. The intake of inulin-type fructans (ITF) at a dose of 8 g/day for 1 year resulted in a significant decrease in fat mass and BMI among non-obese adolescents (Abrams et al., 2007).

The oral intake of probiotics alone or in combination with prebiotics could also decrease the glucose levels in the serum. Various probiotics strains such as Lactobacilli have been used in the animal models such as diet-induced obese/diabetic mice to appraise the effects of these potential probiotics in the amelioration of type 2 diabetes mellitus (T2DM) (Honda et al., 2013). The supplementation with probiotic yogurt (*B. lactis* Bb12 and *L. acidophilus* La5) at a dose of 300 g per day for 6 weeks reduced the fasting blood glucose and glycated hemoglobin (HbA1c) among T2DM patients. Furthermore, probiotics were shown to promote antioxidation among the T2DM patients as an increase in superoxide dismutase and glutathione peroxidase activities were observed in the erythrocytes of the persons supplemented with the yogurt (Ejtahed et al., 2012).

Modification of the fecal microbiota in response to the oral intake of *Lactobacillus* species was seen in some individuals (Goossens et al., 2006; Ferrario et al., 2014). However, studies have also reported that probiotic supplementation does not affect the composition of the fecal microbiota in healthy adults and infants (Kristensen et al., 2016). These inconsistent findings may be due to individual variations in humans, as the consumption of probiotics results in an individualized intestinal colonization pattern that induces a variable response in terms of intestinal microbial population and host metabolism and is controlled by the host’s characteristics (Suez et al., 2018; Zmora et al., 2018). Therefore, it is suggested to further investigate the value of probiotics in modulating the human gut microbiome in both health and disease states with an individualized approach.

## Dietary intervention and host metabolism

There is increasing evidence to suggest that dietary interventions alter host metabolism on an individual basis, primarily due to different gut microbial species as well as host physiology and metabolism (Rowland et al., 2018). The relative abundance of a particular species can predict the host’s response to any specific dietary intervention. For instance, higher levels of *Prevotella* species were associated with better glucose metabolism among healthy individuals after consumption of bread holding barley grains, suggesting the role of *Prevotella* in individualized metabolic enhancement in response to glucose metabolism (Kovatcheva-Datchary et al., 2015). Obese adults who have the highest abundance of mucin-degrading bacteria, i.e., *Akkermansia muciniphila*, showed better lipid metabolism with a greater reduction in body fat after ingestion of a calorie-restricted diet, suggesting the prognostic role of this bacterium in the evaluation of the host response to dietary changes (Dao et al., 2016). Among children with inflammatory bowel disease, individuals with an abundance of Bacteroidaceae, Clostridiales, and Erysipilotrichaceae species responded better to low-fermenting disaccharides and oligosaccharides compared to individuals with higher levels of the genus *Turicibacter* (Chumpitazi et al., 2015). Similarly, individuals with a higher abundance of *Subdoligranulum* and *Sporobacter* and lower levels of *Bacteroides* responded better to the diet holding low-fermentation substrate during the dietary treatment of inflammatory bowel disease in children (Chumpitazi et al., 2014).

Based on the results of dietary interventions, people can be classified into responders and non-responders. To differentiate between responders and non-responders, custom prediction methods have been developed using machine learning approaches that combine various individual traits and baseline microbial diversity. A study that included 800 overweight or obese people has successfully predicted variability in glycaemic response to identical foods by including anthropometric and blood parameters, eating habits, and the gut microbiome. Person-specific predictions were obtained for various dietary components, serum parameters, age, and microbiome. The study suggested that the degree of microbial contribution and clinical and laboratory findings may vary, requiring further investigations for better predictability in different populations (Zeevi et al., 2015). This person-specific approach was confirmed among the non-diabetic population to predict the glycaemic response to the specific food (Mendes-Soares et al., 2019). One study among twins showed a fairly variable interpersonal insulinemic, glycaemic, and lipidemic response to diets, suggesting that the response to the same foods varies even among genetically identical twins. This highlights that host metabolism factors, gut microbiota, nutritional content, meal, times, and exercise play a decisive role in individualized response to diet rather than genetics (Berry et al., 2019). These findings support the idea of personalized nutrition to achieve the same result in different individuals, although this approach and further studies are desired to tailor feasible and sustainable person-specific nutritional strategies to optimize individual gut microbiome and host responses.

## Feeding patterns, microbiota, and host metabolism

**FIGURE 3:**
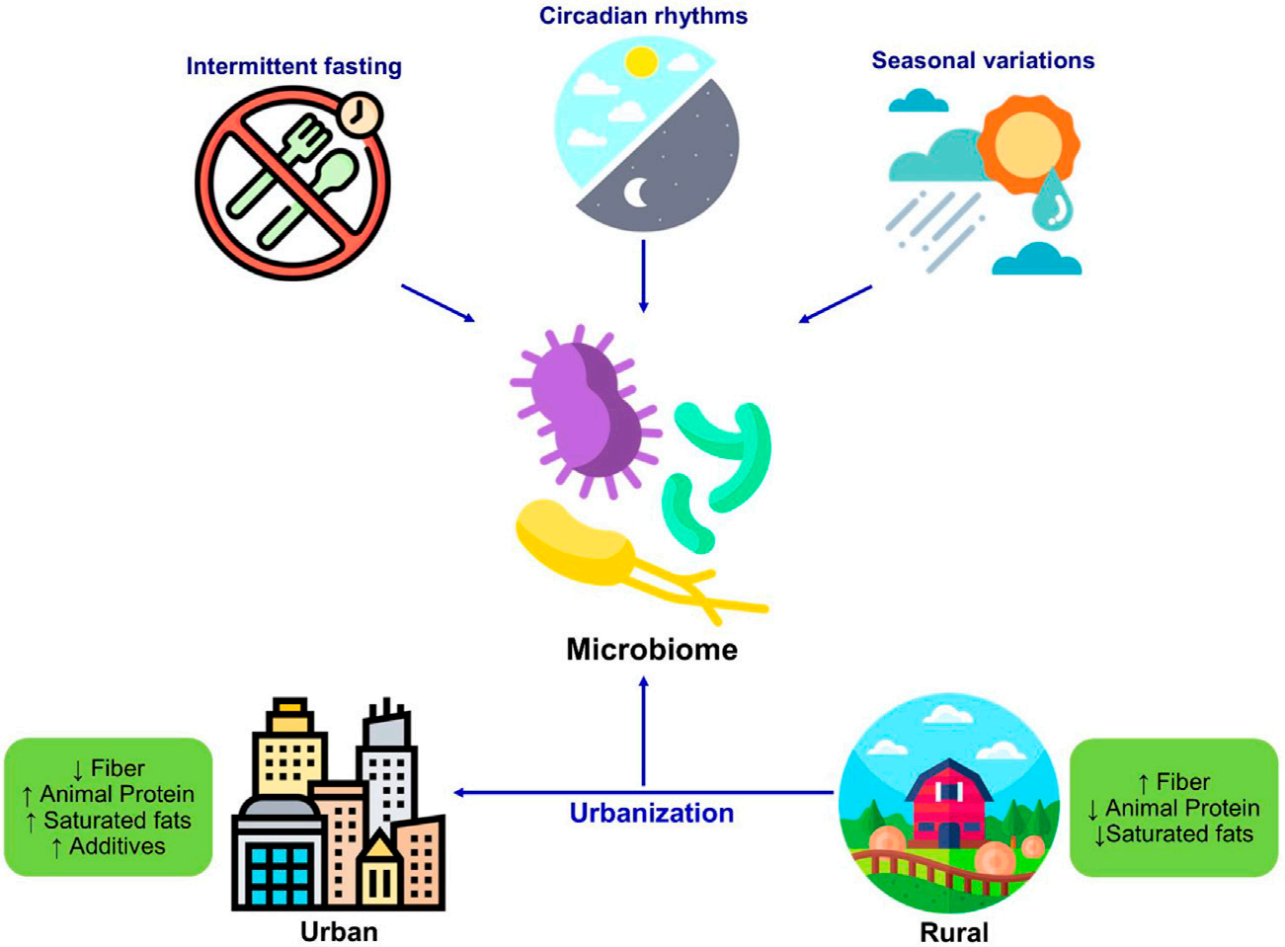
Changes in the microbiome in response to diet and lifestyle. The dietary habits including intermittent fasting seasonal variations, circadian rhythmicity shape the microbiome composition. The westernization resulted in changes in dietary patterns and dietary components which significantly changes the gut microbiome composition as well as function.

The timings of dietary intake, intermittent fasting, and circadian patterns of intake can influence the host metabolism and intestinal microbial population (Figure 3). The rhythm of food intake combined with the circadian clock of the host can affect the circadian fluctuation in the composition and function of gut microbiota in human and mouse models (Mukherji et al., 2013; Thaiss et al., 2014). Therefore, the rhythmicity of microbiota changes in response to the variations in the feeding patterns. For example, microbial diurnal fluctuations are reduced with the intake of a high-fat diet, which sequentially affects the metabolism and circadian clock function in mice (Leone et al., 2015; Sundaram et al., 2020). It is hypothesized that intermittent fasting improves the metabolic health of the host by shaping the gut microbiota (Patterson and Sears, 2017). The study has reported that intermittent fasting in mice alters the composition of gut microbiota and increased lactate and acetate levels, which decreases fat diet-induced obesity (Li et al., 2017). Furthermore, these microbial shifts in response to intermittent fasting also protect humans and mice from multiple sclerosis (Cignarella et al., 2018). Further studies are necessary to explore the role of intermittent fasting in altering the intestinal microbiota and its consequent beneficial effects on other disorders and to evaluate the personalized facets of such interventions.

## Microbiota and metabolic disorders

### Microbial ecology in metabolic disease

The gut is a complex ecosystem that provides resources for the interaction of numerous microbial species. The use of macroecological concepts can be helpful to understand the link between the diversity of gut microbiota and metabolic output (Costello et al., 2012). Improved metabolic health is linked with comparatively increased diversity of microbial species and higher gene content, as clear from the metagenomic studies. The results are constant with the results from the studies focusing on the microbiota among the individual of traditional societies (Cotillard et al., 2013). The studies have highlighted that the microbiota of rural farming populations of different parts of the world was diverse with regard to bacterial species compared to the Western populations (Martínez et al., 2015; Gupta et al., 2017; Senghor et al., 2018). Considerably, some microbial taxa that were found in the gut of traditional peoples, despite living on different continents, were found absent from the Western people. The reason behind this situation is that the diversity of gut microbiota is declining with the loss of certain microbial genera, due to the increasing industrialization of modern lifestyles, the use of processed food, and certain clinical practices. However, it is still unclear whether a varied and healthy diet contributes to increased diversity and whether microbial diversity can be directly helpful in protecting against metabolic disorders. A plausible reason might be the fact that the functional capacity of microbiota is changing among industrialized nations. For example, altered production of SCFAs by the gut microbiota is reported among the population living in industrial nations, which is contributing to certain metabolic disorders including obesity (Forslund et al., 2013; Karlsson et al., 2013). Hence, dietary supplementing, especially the intake of complex carbohydrates, could help to sustain and recover the diverse gut microbiota for an essential set of human body functions. However, it is important to know that the diversity is not only limited to the relative abundance of the microbial population but also the functions encoded in these residents. Further, increased biodiversity is not always associated with health promotion. Therefore, a better understanding of diversity regarding the identity of the organism, functions, and location within the gut can be helpful for the amelioration of such disorders.

### The impact of microbiome metabolites on metabolic disorders

The gut microbiota is continuously producing various small molecules through diverse metabolic pathways (Table 2). The production of most of these molecules may depend on the dietary intake of the host. Some of these molecules are still in the intestine, while many others are absorbed and chemically modified in the systemic circulation and secreted into the urine (Meyer and Hostetter, 2012; Donia and Fischbach, 2015). SCFAs are of special interest due to their diverse role and implications in obesity and metabolic diseases. Elevated levels of SCFA were reported in obese individuals, as well as in animal models, as they were found to offer additional calories to the host (Schwiertz et al., 2010; Cho et al., 2012). Four different signaling pathways of these compounds have been reported in the host. First, SCFAs, especially butyrate, act as an energy substrate for the epithelial cells of the colon. In germ-free (GF) mice, transit was slow in the small intestine in response to decreased energy availability to provide a longer duration of nutrient absorption (Donohoe et al., 2012; Wichmann et al., 2013). Second, SCFAs i.e., propionate can induce gluconeogenesis in the gut that can protect the human host from glucose intolerance and diet-induced obesity (Jang and Lee, 2021). Third, SCFAs, such as acetate and butyrate, can function as histone deacetylase (HDI) inhibitors involved in cell cycle arrest and apoptosis (Davie, 2003). Finally, SCFAs signal through G-protein-coupled receptors (GPCRs), for example, GPR41 and GPR43, which affect several key processes, such as enteroendocrine regulation and inflammation (Brentani, 1988; Samuel et al., 2008).

**TABLE 2:** The implications of microbiota-derived metabolites in metabolic diseases.

Metabolites	Type	Metabolic role	Study/intervention type	References
Acetate	SCFAs	Decreased appetite and intake of nutrition	Mice	Frost et al. (2014)
Butyrate	SCFAs	Improves energy metabolism and decreases insulin resistance	Mice	Li et al. (2018)
Ceramide	Lipids	Decreased cold-induced thermogenesis	Mice	Zhang et al. (2019)
Ethanol	—	Epithelial tight junction dysfunction	Humans	Rao et al. (2004)
Glycodeoxycholic acid	Bile acids	Decreased insulin resistance	Mice	Qi et al. (2019)
Glycoursodeoxycholic acid	Bile acids	Decreased hyperglycemia	Mice	(Sun et al., 2018)
Histamine, spermine and Taurine	—	Increased IL-18	Mice	(Levy et al., 2015)
10-hydroxy-cis-12-octadecenoic acid (HYA)	Octadecenoic acid	Decreased obesity	Mice	(Miyamoto et al., 2019)
Indole-3-aldehyde	—	Increased IL-22	Mice	(Zelante et al., 2013)
Propionic acid	SCFAs	Increased expression of leptin mRNA	Human explants	(Al-Lahham et al., 2010)
Propionate and butyrate	SCFAs	Regulation of energy intake and insulin secretion	Human enteroendocrine cell lines	(Larraufie et al., 2018)
Propionate and butyrate	SCFAs	Release of glucagon-like peptide-1 (GLP1) and peptide YY (PYY)	Rats	(Psichas et al., 2015)
Tauro-beta-muricholic acid	Bile acids	Decreased glucose intolerance	Hamsters	(Sun et al., 2019)
Tauroursodeoxycholic acid	Bile acids	Decreased insulin resistance	Humans	(Kars et al., 2010)

Microbes metabolize phosphatidylcholine, a phospholipid, and L-carnitine, an amino acid found in food, to produce trimethylamine (TMA) (Koeth et al., 2013). TMA is absorbed from the intestine into the blood and is transferred to the liver for enzymatic oxidation to trimethylamine N-oxide (TMAO). TMAO has been implicated in people at increased risk for cardiovascular disorders and increased atherosclerosis in mouse models (Koeth et al., 2013; Tang et al., 2013). The production of TMA is an excellent example that describes the interface between the microbiota and the diet. In those cases, when the microbiota can produce TMA, the metabolite is only produced if the diet holds the substances that contain trimethylammonium compounds. The microbiota among the persons on a vegetarian diet is often poor producers of TMA, even if their diet is temporarily fortified with the precursor compounds (Koeth et al., 2013). The data suggest the evolution of the microbiota to adapt to the specific types of macronutrients present in the diet. Experiments have shown that TMAO participated in promoting atherosclerosis in animals supplemented with low-fat diets and the compound. The overall metabolism was improved, and the risk of cardiovascular disorders was reduced in people who underwent weight-loss surgeries but with a higher level of TMAO in the blood. Among these patients, the elevated circulating TMAO could be attributed to the aerobic environment of the intestine that is favorable for the generation of these compounds. Therefore, the conditions that favor TMAO-causing cardiovascular disease should be further explored in humans (Sjöström et al., 2007; Sjöström et al., 2012).

## Microbiota and personalized nutrition

Personalized nutrition may not be limited to metabolic disorders and may be extended as supportive therapy for immune diseases, especially those related to the gut, cancer, and neurological disorders, as well as prophylactic therapy for individuals at high risk of disorders related to lifestyle or blood. genetics. The diet, and especially the types of consumed polysaccharides, modulate the general composition as well as the function of the intestinal microbiota. The low cost, availability, and relative safety of polysaccharides make them an interesting food with beneficial health properties, but the precise concentration of individual polysaccharides or combinations must be determined to improve human health.

The modulation of gut microbiota is not straightforward as the individuality of composition is quite high. Among a cohort of around three thousand people, a total of 664 genera were revealed and only 14 of these genera were found in 95% of the people (Falony et al., 2016) The composition of the microbiota in the adult gut varies among the individuals with huge differences in terms of presence and the absolute and relative numbers of genera (Vandeputte et al., 2017). For diet selection, the rational approach, as well as machine learning approaches, can be helpful. For rational design, microbiome signatures with their metabolic properties are identified. When the microbial population is classified, beneficial foods for all types of microbiota can be identified to obtain the desired results. The machine learning approach is best for complex traits and does not require prior knowledge of complex mechanisms and can therefore be used for any measurable characteristic (Zeevi et al., 2015). In one study, blood glucose was monitored for postprandial glycemic responses among 800 people using a machine-learning approach. Predictions related to the response of individuals to the given food were predicted and confirmed (Zeevi et al., 2015). Likewise, variations in glucose metabolism were seen among individuals in response to dietary fiber. Glucose tolerance improved among individuals with an abundance of the genus *Prevotella* in the gut microbiota (Kovatcheva-Datchary et al., 2015). These studies point to the perspective of these approaches to decide on the dietary intervention that could be suitable for any individual or population for the improvement of pathological conditions.

## Conclusion and future prospects

The correlation between diet type, microbiota, and metabolites can be challenging. The use of probiotics and prebiotics can help regulate the microbiota-diet axis and change the composition of the microbiota for better results combined with personalized dietary regimens. Most of the studies so far have correlated the interactions between the microbiota, food, and host metabolism, and some of them have described the mechanisms involved in the interactions of these three components. Furthermore, the mechanisms involved in the interaction between these three entities are concluded from mouse models that are different in their physiology and metabolism compared to humans (Beura et al., 2016). Wider individual variability among humans, difficulties in controlling microbiota composition, and host compliance with the experimental diet are some of the major challenges (Kolodziejczyk et al., 2019). Large cohort follow-up studies are particularly recommended to study nutritional interventions and amelioration of metabolic disorders, for which experiments must last longer periods, which seems impractical.

All three contributors—the microbiota, food, and human metabolism—are quite complex and have unique kinds of limitations. For example, the characterization of microbial species is associated with technical difficulties and disparities such as sample storage conditions, DNA extraction protocols, and methods used to construct sequencing libraries. Furthermore, only a fraction of the genes encoded by microbial species in the gut are known and the functions of most of them are predicted based on sequence similarity. To cite an example, the function of only 65% of *Escherichia coli* genes has been determined, despite being the most studied bacterium (Ghatak et al., 2019). This percentage is much lower for other bacteria, especially those that are difficult to grow. In addition, bacterial metabolism is being studied through *in vitro* functional studies, generally in monocultures, which is very different from the intestinal environment of the host intestinal tract and therefore does not portray a clear image of the network formed by the intestinal and microbial species. The use of mass spectrometry for the identification of metabolites also presents disparities due to differences in sample preparation and extraction and methodological variations (Patti et al., 2012).

Computational tools are being adopted to overcome these complications and the algorithms employed rely on data such as the amount and composition of the diet, the human response, and the composition of the microbiome to predict the general effects of these factors on the expected results. The limitation of personalized nutrition studies is that the studies are conducted in populations of Western countries that consume the Western diet, so the findings may not be generalized to other societies that consume different products. In addition, to achieve the goal of individualized nutrition, the design of an optimal diet is not enough, and the support of people to ensure compliance and support remains crucial. Despite several limitations, advances in microbiome research are promising for the design of comprehensive studies and the application of computational tools in the analysis of large data sets to design a personalized diet for the improvement of particular clinical conditions.

The association between altered microbiota and metabolic diseases is becoming clear in studies using animal models and human subjects ranging from obesity to T2DM and cardiovascular disease. In order to move forwards, a clear understanding of how much the gut microbiota is linked to the metabolism through large cohort studies with a substantial number of participants. The transfer of microbiotas from human subjects to mice can be a potential approach, especially when employed on twin cohorts. It is also important to know about the role of diet in the amelioration of disease states that are linked with microbiota alterations and to understand the underlying molecular mechanisms. The fecal microbiota transplantation that has been demonstrated to cure the recurrent infections caused by *Clostridium difficile* can also be used to study the role of gut microbiota in host metabolism. In the future, a better interpretation of the mechanistic basis of personalized nutrition and the simplification of these approaches to extend the range for large populations are essential to help nutritionists make rational use of diet in the prevention and treatment of human diseases.
